# Could DOAC Be a Better Choice Than Warfarin in Low Compliance Patients with Fontan Procedure?

**DOI:** 10.3390/medicina57050465

**Published:** 2021-05-10

**Authors:** Jisoo Park, Bo-Young Hong, Joon-Sung Kim, Jung-Geun Park, Jiyoon Jung, Seong-Hoon Lim

**Affiliations:** Department of Rehabilitation Medicine, St. Vincent’s Hospital, College of Medicine, The Catholic University of Korea, Seoul 07345, Korea; sallypjs@hanmail.net (J.P.); byhong@catholic.ac.kr (B.-Y.H.); svpmr@chol.com (J.-S.K.); jgp0123@naver.com (J.-G.P.); jennylidada@gmail.com (J.J.)

**Keywords:** Fontan procedure, thromboembolic events, cerebral infarct, pulmonary thromboembolism, renal infarct, warfarin, DOACs, dabigatran

## Abstract

*Background and Objectives:* After the Fontan procedure, thromboembolic events need to be prevented. We present a young patient with a history of Fontan procedure and poor adherence to warfarin who developed systemic thromboembolism. He was changed to maintenance dabigatran, which is one of the available direct oral anticoagulants (DOACs). *Case presentation:* A 20-year-old man was diagnosed with cerebral infarct, pulmonary thromboembolism (PTE), and renal infarcts. He was prescribed warfarin to prevent thromboembolic events after the Fontan procedure. Based on his poor adherence to warfarin, we decided to change the anticoagulant therapy from warfarin to dabigatran 150 mg bid. One month later, his pulmonary thromboembolism regressed. *Conclusion:* Our case report showed a young adult with low compliance to warfarin who developed cerebral, pulmonary, and renal thromboembolic events. Thus, in our opinion, the change from warfarin to a DOAC was necessary for further prevention and treatment of PTE. A change from warfarin to a DOAC should be considered in patients with poor compliance who are at high risk of thromboembolic events, for example, after the Fontan procedure.

## 1. Introduction

Fontan procedure is the treatment of choice for newborns with one functioning cardiac ventricle. The surgeon modifies the vasculature by creating pathways to separate oxygenated and deoxygenated blood and permit a single ventricular pump. The reported incidence of thrombosis and embolism after the Fontan operation ranges from 4% to 20% and occurs due to impaired blood flow in the non-pulsatile circuit, presence of prosthetic material, right-to-left shunts, blind cul-de-sacs, atrial arrhythmias, and altered coagulation. However, many patients are asymptomatic [1]. The difficulty in monitoring and regulating warfarin use usually has been for young patients [2]. Thus, there is no evidence that anticoagulation with warfarin is superior to antiplatelet therapy for preventing thromboembolic events in post-Fontan procedures [2].

In 2019, the American Heart Association/American College of Cardiology/Heart Rhythm Society strongly recommended the use of direct oral anticoagulants (DOACs) for patients who have an indication for either warfarin or a DOAC [3]. Direct thrombin inhibitor (dabigatran) and factor Xa inhibitors (rivaroxaban, apixaban, and edoxaban) are now the standard treatments for preventing stroke and systemic embolism in patients with atrial fibrillation and for venous thromboembolism [3]. However, in patients with mechanical valve replacement, the use of dabigatran was associated with a higher incidence of thromboembolism and hemorrhage, and warfarin or acenocoumarol should be used in these patients [4].

Compliance is lower with warfarin (a vitamin K-dependent antagonist oral anticoagulant) than DOACs, and fewer than 50% of stroke patients prescribed warfarin continue using it for two years [5]. Another study showed general adherence to vitamin K-antagonists and DOACs were suboptimal [6]. The adherence to anticoagulants is important for preventing thromboembolic events. DOACs have fewer interactions with both food and drugs since some of them are relevant [7]. We present a young patient with a history of Fontan procedure and poor adherence to warfarin who developed systemic thromboembolism. In this case, we replaced the warfarin with dabigatran.

## 2. Case Report

**Figure 1:**
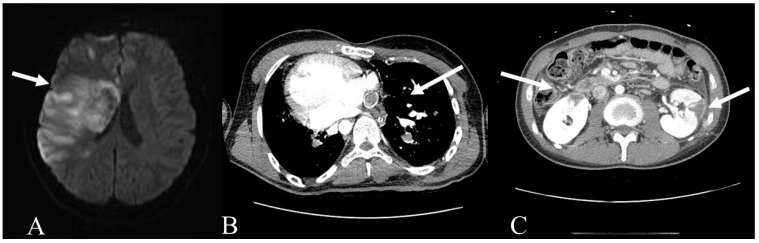
Multiple embolic events occurred in our patient, including (**A**) acute right middle cerebral artery territory infarction on magnetic resonance imaging, (**B**) pulmonary thromboembolism, and (**C**) multifocal renal infarctions on computed tomography.

A 20-year-old man who suddenly developed drowsiness, aphasia, and left hemiplegia was admitted to the hospital. He underwent the Fontan operation as a newborn, as he had an atrioventricular septal defect, transposition of the great arteries, and dextrocardia. He was prescribed oral warfarin to prevent thromboembolic events while maintaining an international normalized ratio (INR) of 2–3. Recently, he stopped taking warfarin consistently; moreover, there was no routine INR monitoring. The initial INR was 1.46, a daily dose of warfarin of 1.5 mg. Imaging studies revealed right middle cerebral artery infarction, pulmonary thromboembolism in the main bronchial artery, and bilateral multifocal renal infarctions (Figure 1). Based on his poor adherence to warfarin, we decided to switch him to dabigatran 150 mg bid. One month later, his pulmonary thromboembolism had regressed. He recovered neurologically via intense rehabilitation and was referred to a clinic for long-term inpatient rehabilitation.

## 3. Discussion

The indications for and complications of warfarin are well known. However, vitamin K-dependent antagonist oral anticoagulants have narrow therapeutic windows and many interactions, thus necessitating strict regulation of diet, regular INR monitoring, and dosage control. Poor adherence to anticoagulants or antiplatelets can lead to thromboembolic events regardless of the primary disease requiring thromboembolism prevention [5]. For the prevention of thrombosis after the Fontan procedure, the anticoagulant should be chosen according to the individual’s risk of thrombosis. Options include warfarin, aspirin, and low-molecular-weight heparin [8]. A clinical trial (the UNIVERSE study) is currently comparing rivaroxaban, a direct factor Xa inhibitor, and acetylsalicylic acid for thromboprophylaxis in post-Fontan children under the age of 8 years [9]. Our patient was a young adult with low warfarin compliance who had multiple thromboembolic events in the brain, pulmonary arteries, and kidneys. Based on his poor adherence to warfarin, we decided to change the anticoagulant therapy from warfarin to dabigatran 150 mg bid.

Young patients treated with a DOAC have a much lower risk of hemorrhage. In 2017, a single-center study of DOACs examined 21 post-Fontan-procedure thromboembolism patients, which is the largest patient group studied to date. The HAS-BLED, which covers common issues in adult Fontan patients, including renal and liver disease and INR lability, and predicts the hemorrhagic transformation rate, was low in this group as most of the patients were young, as in the case of our patient [10]. The study showed that 21 patients over a total of 316 cumulative months tolerated DOACs therapy with 1 new thrombotic event, no major or nonmajor bleeding events, and 10 patients experienced 11 unique minor bleeding events, suggesting that DOACs are an alternative to traditional anticoagulation [10].

## 4. Conclusions

DOACs could be considered in patients with systemic thromboembolism, in whom the INR is poorly controlled after the Fontan operation. Studies including more patients are necessary to determine whether a DOAC, aspirin, or warfarin is the best option for preventing systemic thromboembolism, with consideration of compliance, in patients undergoing the Fontan operation.

## Data Availability

The datasets generated during and/or analyzed during the current study are available from the corresponding author on reasonable request.
